# *GPR143* Gene Mutations in Five Chinese Families with X-linked Congenital Nystagmus

**DOI:** 10.1038/srep12031

**Published:** 2015-07-10

**Authors:** Ruifang Han, Xiaojuan Wang, Dongjie Wang, Liming Wang, Zhongfang Yuan, Ming Ying, Ningdong Li

**Affiliations:** 1 Tianjin Medical University, Tianjin, 300070, People’s Republic of China; 2 Molecular Genetic Department, Tianjin Eye Hospital, Tianjin Eye Institute, Tianjin Key Lab of Ophthalmology and Visual Science, Tianjin, 300020, People’s Republic of China; 3 Ophthalmologic Department, Xuzhou Eye Institute, Xuzhou, Jiangsu Province, 221000, People’s Republic of China; 4 Ophthalmologic Department, Jinan Central Hospital Affiliated to Shandong University, Jinan, Shandong Province, 250013, People’s Republic of China

## Abstract

The ocular albinism type I (OA1) is clinically characterized by impaired visual acuity, nystagmus, iris hypopigmentation with translucency, albinotic fundus, and macular hypoplasia together with normally pigmented skin and hair. However, it is easily misdiagnosed as congenital idiopathic nystagmus in some Chinese patients with OA1 caused by *the G-protein coupled receptor 143* (*GPR143*) gene mutations. Mutations in *the FERM domain–containing 7* (*FRMD7*) gene are responsible for the X-linked congenital idiopathic nystagmus. In this study, five Chinese families initially diagnosed as X-linked congenital nystagmus were recruited and patients underwent ophthalmological examinations. After direct sequencing of the *FRMD7* and *GPR143* genes, five mutations in *GPR143* gene were detected in each of the five families, including a novel nonsense mutation of c.333G>A (p.W111X), two novel splicing mutations of c.360+1G>C and c.659-1G>A, a novel small deletion mutation of c.43_50dupGACGCAGC (p.L20PfsX25), and a previously reported missense mutation of c.703G>A (p.E235K). Optical coherence tomography (OCT) examination showed foveal hypoplasia in all the affected patients with nystagmus. Our study further expands the *GPR143* mutation spectrum and contributes to the study of *GPR143* molecular pathogenesis. Molecular diagnosis and optical coherence tomography (OCT) are two useful tools for differential diagnosis.

Congenital nystagmus (CN) refers to a group of ocular motor disorders characterized by rapid to-and-fro oscillations of the eyes, which presents at birth or develops after 3 ~ 6 months. It may be classified clinically as “sensory defect type ” or “motor defect type”^1^. Sensory defect nystagmus is usually secondary to the afferent disturbance of visual signal from the fovea to the brain, and may be caused by such ocular diseases as albinism, congenital cataract, Leber’s congenital amaurosis, and anterior segment dysgenesis. Motor defect type could be caused by the efferent signal disturbance from the ocular motor control center in the brain to the ocular muscles. As this type is not combined with disorders of the eyes and visual pathways, motor defect nystagmus is usually called as “congenital idiopathic nystagmus” or “infantile nystagmus”.

Congenital nystagmus has a Mendelian inheritance pattern with the X-linked form the most common^2^. Currently, three loci associated with X-linked congenital nystagmus have been mapped on the X-chromosome, where NYSTAGMUS 1 (NYS1) is located at Xq26–q27 (OMIM #310700), NYS5 at Xp11.4–p11.3 (OMIM %300589), and NYS6 at Xp22.3–p22.2 (OMIM #300814).*The G-protein coupled receptor 143* (*GPR143*) and *the FERM domain–containing 7* (*FRMD7*) genes are two disease-causing genes identified from the regions of Xp22.3–p22.2 and Xq26-q27 respectively. *FRMD7* gene mutations have been documented to be the cause of congenital idiopathic nystagmus^3^, while *GPR143* gene mutations are believed to be responsible for ocular albinism type I (OA1)^4^.

The typical clinical features of the OA1 include impaired visual acuity, nystagmus, iris hypopigmentation with translucency, albinotic fundus, and macular hypoplasia together with normally pigmented skin and hair. As nystagmus can be the prevalent feature of OA1, it is easily misdiagnosed as congenital idiopathic nystagmus, especially for those patients without typical iris hypopigmentation and albinotic fundus changes. Molecular diagnosis and optical coherence tomography (OCT) are two useful tools for differential diagnosis. However, the treatment principle for OA1 is the same as that for congenital idiopathic nystagmus.

Here we describe five Chinese families with X-linked congenital nystagmus (CN). Molecular genetic analysis of the two candidate genes (*FRMD7* and *GPR143*) indicated that five mutations in *GPR143* were detected in these families. Macular hypoplasia was found by OCT in the patients with *GPR143* mutations.

## Patient Ascertainment

**Figure 1:**
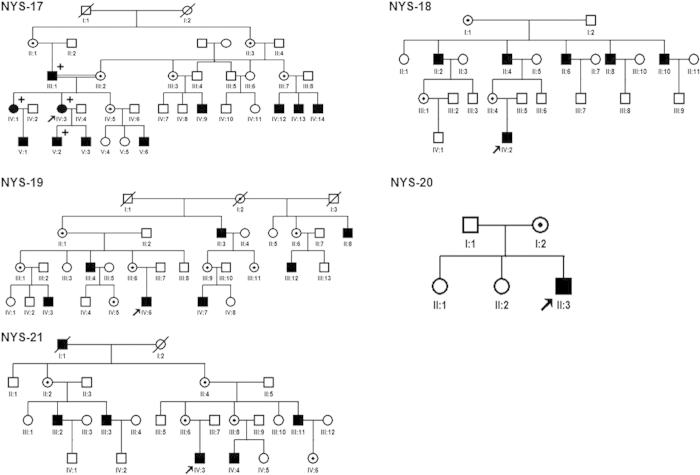
Five Chinese families with X-linked OA1. The squares represent males, the circles represent females, the shaded symbols indicate the affected individuals, the dotted circles represent the female carriers, a diagonal line through a symbol indicates a deceased family member, and the arrow indicates the proband. The plus sign indicates the individual who diagnosed as congenital nystagmus combined with exotropia.

Five families with X-linked congenital nystagmus (NYS-17, NYS-18, NYS-19, NYS-20, and NYS-21) were recruited for this study (Fig. 1). All participants underwent an ophthalmologic examination, including best corrected visual acuities, anterior segment of the eyes, vitreous and fundus, OCT, Visual Evoked Potential (VEP) and electroretinography (ERG). After informed consent, 25 affected and 34 unaffected individuals from these five unrelated families had 3 ml blood samples taken and DNA was extracted from blood lymphocytes according to standard protocols (Roche Biochemical, Inc). This study obtained the institutional review board (IRB) approval from the Tianjin Eye Hospital and conformed to the tenets of the Declaration of Helsinki.

### Mutation Analysis

The sequence analysis of *FRMD7* and *GPR143* was carried out by direct genomic DNA sequencing after PCR amplification of whole coding regions and exon-intron boundaries of these two genes using the previously published primers^5^. PCR was carried out in 20 μL of standard PCR buffer containing 1.5 mM MgCl_2_, 0.2 mM of each dNTP, 0.5 μM of each primer, 1 U of Taq polymerase (Sangon, Shanghai, China), and 50 ng of DNA. The amplification program was an initial 2 min denaturation at 98 °C, followed by 30 cycles of 30 s at 94 °C, 30 s at 55 °C, 1 min at 72 °C, and a final 7 min extension step at 72 °C. The PCR products were bi-directionally sequenced using the BigDye Terminator Cycle Sequencing V3.1 kit on an ABI PRISM 3130 Genetic Analyzer (Applied Biosystems) after purification with the QIAquick Gel Extraction Kit (Qiagen, Valencia, CA). Sequencing results were assembled and analyzed with the Seqman program of DNASTAR software (DNASTAR Inc, Madison, WI). Mutation naming followed the nomenclature recommended by the Human Genomic Variation Society (HGVS). Splicing mutations were analyzed on line using the Human Splicing Founder Server^6^ (http://www.umd.be/HSF/).

### Reverse transcription PCR and real-time quantitative PCR

As we only obtained RNA from the NYS-17 family, real-time quantitative PCR was performed to analyze the *GPR143* gene dosage change in this family. Total RNA was prepared from venous blood (0.2 ml) from the affected male individuals, the female carriers and the normal controls, using the RNA isolation kit for mammalian blood (Sangon, Shanghai, China). One microgram of total RNA from each sample was reverse transcribed into cDNA. The primer sequences for the *GPR143* gene were CCGTGTGGTTAGGATTCCC (forward) and CCCACGCCATGATGTGATAC (reverse). As an endogenous control, the *GAPDH* gene with the primer sequences CCAAAATCAAGTGGGGCGAT (forward) and TGATCTTGAGGCTGTTGTCA (reverse) was used. For amplification of *GPR143* and *GAPDH*, PCR reactions conditions were 95 °C, 3 min; 95 °C, 30 s; 58 °C 30 s and 72 °C, 1 min for 35 cycles with a final elongation step at 72 °C for 5 min. Real-time quantitative PCR was performed using the SYBR Premix ExTaq kit (Takara, Dalian, China) in accordance with the manufacturer’s instructions. The PCR cycling conditions were as follows: 95 °C for 1 min, followed by 40 cycles of 95 °C for 15 s and 60 °C for 40 s. Real-time quantitative PCR was performed using Eppendorf Mastercycler ep realplex real-time PCR system. The cycle threshold (C_T_) values were obtained from each PCR reaction. Data analyses were performed using the relative quantitative method (2^−ΔΔCT^) for calculating relative gene expression. ΔΔC_T_ = ΔC_T_ sample – ΔC_T_ control.

## Results

### Clinical features

**Figure 2:**
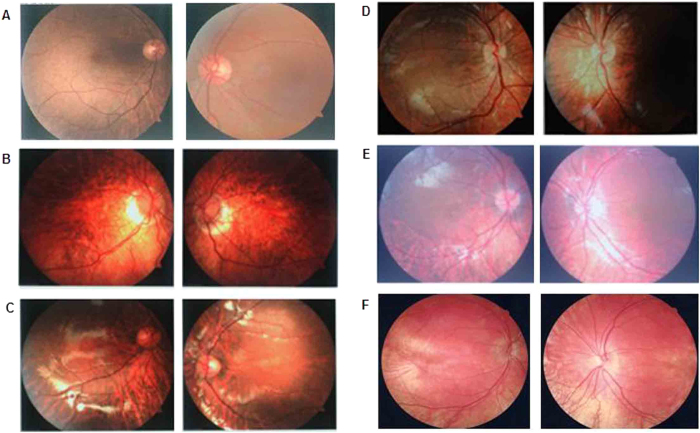
Photographs of the patients’ fundus appearance. (**A**) is the fundus photograph of a normal control. (**B**) is the fundus photograph of individual IV:3 (NYS-17) showing myopia change and hypopigmentation in the posterior of the fundus. (**C**), (**D**), (**E**) and (**F**) are the fundus photographs of individual IV:2 (NYS-18), individual IV:6 (NYS-19), individual II:3 (NYS-20) and individual IV:3 (NYS-21) showing variable hypopigmentation in the fundus.

**Figure 3:**
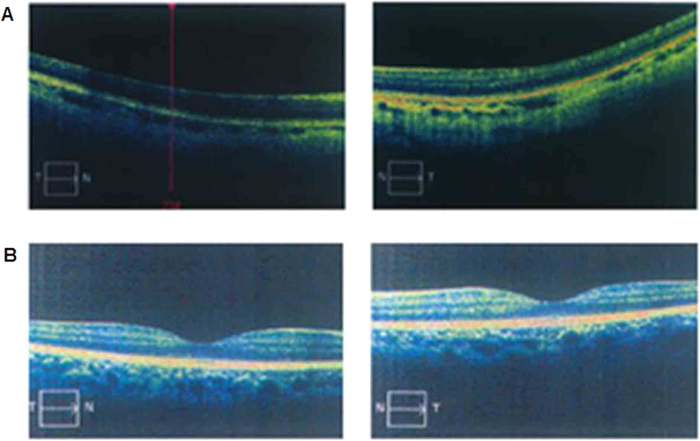
Photographs of OCT examination. Compared with a normal control (**B**), the patients (**A**) carrying with *GPR143* gene mutations showed macular hypoplasiain OCT examination.

All the families (NYS-17, NYS-18, NYS-19, NYS-20, and NYS-21) were referred to Tianjin Eye Hospital for congenital nystagmus. We evaluated the clinical features for 9 patients and 5 female carriers in these five families (Table 1). The most common clinical features among the patients were nystagmus, impaired visual acuity, variable hypopigmentation in the fundus and macular hypoplasia detected by OCT. However, iris hypopigmentation with translucency was not evident. With the exception of the proband in the NYS-18 family, having a chin-down head position to compensate his vertical nystagmus, other probands in the NYS-17, NYS-19, NYS-20, and NYS-21 families showed a horizontal pendular nystagmus in the primary eye position without a compensative head position. In addition, exotropia was one of the clinical features in the NYS-17 family. The NYS-17 family was a large highly consanguineous family including 11 patients and 6 female carriers (Fig. 1). The individual III:1 was a 65-year-old male diagnosed with congenital nystagmus combined with exotropia. He married his cousin (individual III:2), a female carrier without nystagmus and exotropia. In his family, two of his daughters (individual IV:1 and individual IV:3) were affected patients with nystagmus and exotropia, and one of his daughters was a female carrier (individual IV:5) without nystagmus and exotropia. The individual IV:3 was the proband in the NYS-17 family and diagnosed with congenital nystagmus combined with exotropia. She developed nystagmus at the age of 3 to 4 months, and had horizontal pendular nystagmus and exotropia of 20° in the primary eye position with best corrected visual acuity of 0.1 in both her eyes. Photographs of her fundus showed hypopigmentation in the posterior of the fundus and allowed visualization of the choroidal vessels (Fig. 2). The OCT documented macular hypoplasia and thinning of her retina (Fig. 3).

### Identification of Mutations

**Figure 4:**
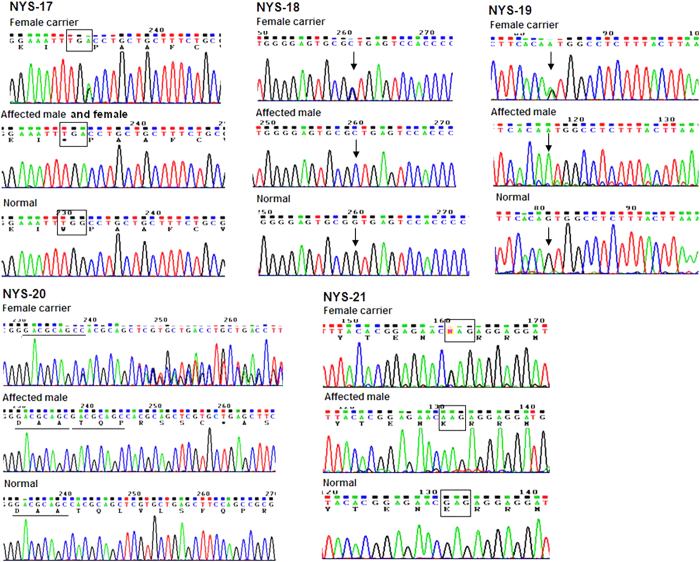
Sequence analysis of *GPR143* gene for five families with congenital nystagmus. Sequencing chromatograms from a female carrier (top), an affected male (middle) and a normal individual (bottom) in each family. Mutations in *GPR143* were identified in each of five families: c.333G>A (p.W111X) in the family NYS-17, c.360+1G>C in the family NYS-18, c.659-1G>A in the family NYS-19, c.43_50dupGACGCAGC (p.L20PfsX25) in the family NYS-20, and c.703G>A (p.E235K) in the family NYS-21.

**Figure 5:**
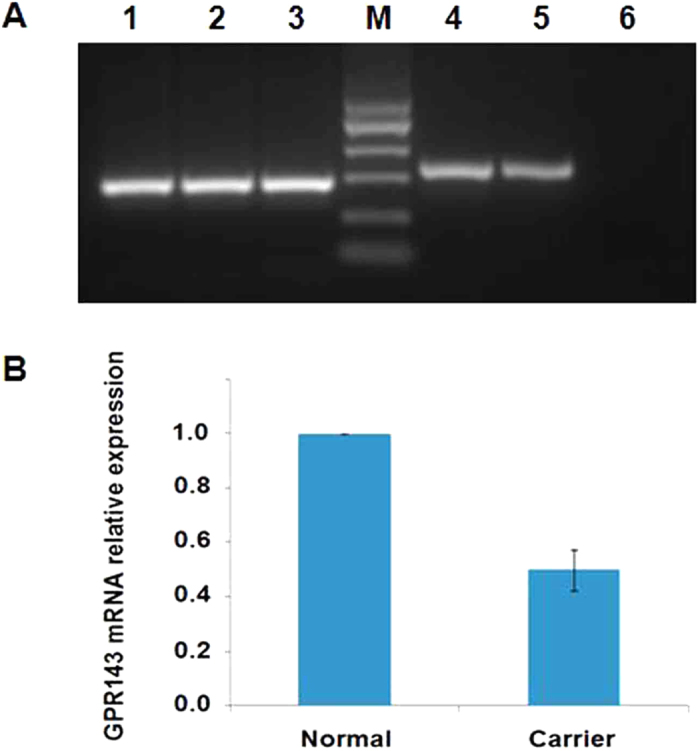
Reverse transcription PCR and real-time quantitative PCR analysis of *GPR143* mRNA expression in Family NYS-17. (**A**). Reverse transcription PCR analysis of *GPR143* mRNA expression in Family NYS-17.The results showed that the *GAPDH* transcripts were detected in a normal control, a female carrier and an affected male (Lane 1, 2, and 3, 192 bp size). However, the target product of a 219 bp size by RT-PCR amplification from *GPR143* was absent in the affected male (Lane 6), but present in the normal control (Lane 4), and the female carrier (Lane 5). M: the Marker ladder of 25 bp, 100 bp, 200 bp, 300 bp, 400 bp, 500 bp. (**B)**. The quantitative gene expression in the female carrier and the normal control. The level of *GPR143* transcripts of the female carrier was about one-half of the normal control level. The 2^−△△Ct^ relative quantitative method was used to analyze the level of *GPR143* mRNA expression in the female carrier compared with the normal control. The standard errors were shown in column chart. (The electrophoretic gel of Figure 5A was cropped, and the full-length gel was presented in supplementary figure 1).

After sequence analysis of the *GPR143* and *FRMD7* genes, we found a hemizygous mutation of c.333G>A (p.W111X) in exon 2 of *GPR143* in all affected males in the NYS-17 family, and a homozygous mutation of c.333G>A (p.W111X) in the affected females (individualIV:1 and individual IV:3), and a heterozygous mutation of c.333G>A (p.W111X) in all female carriers (Fig. 4). Real-Time quantitative PCR revealed that the level of *GPR143* transcripts of the female carriers were about one-half the control level (Fig. 5).

Two potential splicing mutations, c.360+1G>C and c.659-1G>A in *GPR143*, were detected in the families of NYS-18 and NYS-19 respectively. The Human Splicing Founder Server program predicted that these two splicing mutations would produce a consensus value (CV) variation score of –29.06 for c.360+1G>C and –32.5 for c.659-1G>A respectively, and lead to skipping of the wild type splice site.

**Figure 6:**
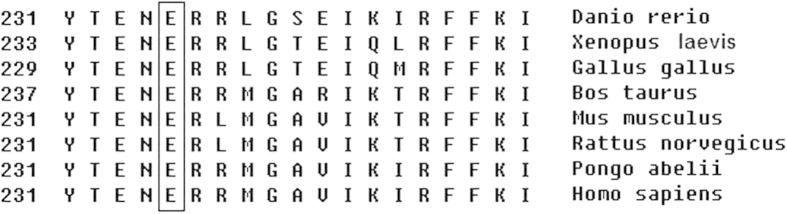
Alignment of *GPR143* amino acids. The alignment of amino acids around p.E235K (denoted by the black framework) of *GPR143* revealed evolutionary conservation of the Glutamic acid among *Homo sapiens, Pongo abelii, Rattus norvegicus, Mus musculus, BosTaurus, Gallus gallus, Xenopus laevis, and Danio rerio*.

In family NYS-20, an 8 bp duplication c.43_50dupGACGCAGC (p.L20PfsX25) in exon 1 of *GPR143* was identified in the affected male and his mother. A previously reported missense mutation c.703G>A (p.E235K)^7^ was detected in exon 6 of *GPR143* in NYS-21 family. The multiple sequence alignment of the *GPR143* protein shows that E235 was conserved among *Homo sapiens*, *Pongo abelii*, *Rattus norvegicus, Mus musculus*, *Bos taurus, Gallus gallus*, *Xenopus laevis*, and *Danio rerio* (Fig. 6). A position-specific independent count (PISC) score of 1.0 was obtained after using the POLYPHEN program (http://coot.embl.de/PolyPhen/) to predict the functional and structural changes of the amino acid substitution. All the mutations described above were absent in 100 normal controls. Sequence analysis of the *FRMD7* gene showed that no mutations were detected in these five families.

## Discussion

In this study, we describe the clinical characteristics of five Chinese families with X-linked nystagmus, and have identified four novel mutations in these families, c.333G>A (p.W111X), c.360+1G>C, c.659-1G>A and c.43_50dupGACGCAGC (p.L20PfsX25), and a known mutation c.703G>A (p.E235K) in these families.

The *GPR143* gene consists of 9 exons spanning approximately 40 kb on chromosome Xp22.3, and encodes a protein of 404 amino acids. It is highly expressed in the retinal pigment epithelium (RPE) and melanocytes. The GPR143 protein contains seven putative transmembrane domains, and is localized to RPE melanosomal membranes. It functions as a receptor of tyrosine, L-DOPA and dopamine, playing an important role in melanosome biogenesis, organization and signal transduction^8^,^9^. To date, more than one hundred mutations in *GPR143* have been reported to be responsible for ocular albinism type I (Human Gene Mutations Database http://www.hgmd.cf.ac.uk). However, the exact molecular mechanism of how the mutated *GPR143* causes ocular abnormalities, such as nystagmus and macular hypoplasia, is still unknown. It has been proposed that GPR143 could be involved in melanosomal biogenesis as a ligand of L-DOPA, a precursor in melanin synthesis. Mutations in *GPR143* could affect melanin synthesis in the retinal pigment epithelium (RPE), and result in abnormal development of the retina and visual pathways, which could be the cause of the misrouting of the optic fibers, nystagmus, foveal hypoplasia, and reduced visual acuity^10^,^11^,^12^,^13^,^14^.

The nonsesnse mutation of c.333G>A (p.W111X) in exon 2 occurred in the transmembrane domain of the GPR143 protein and alters the length of the open reading frame, creating an aberrant truncated protein because of a wild type amino acid of tryptophan (Try, W) at codon 111 substituted by a premature termination codon (PTC). The nonsense-mediated mRNA decay (NMD) mechanism could be activated to degrade mRNA with a PTC in order to prevent the truncated harmful protein interfering with normal cellular processes^15^. We have demonstrated in this study that the *GPR143* mRNA level was about 50% lower in female carriers than in normal controls in the family NYS-17.

The splicing mutation c.360+1G>C in *GPR143* occurred in the 5′ consensus donor region for the splicing of intron 2–3, while the splicing mutation c.659-1G>A occurred in the 3′ consensus acceptor region for the splicing of intron 5–6. Both these splicing mutations would be predicted to result in loss of the original splicing site, exon skipping, activation of a cryptic splice site and introduction of a premature termination codon (PTC)^6^. Using the online tools of HSF program and ORF finder (https://www.ncbi.nlm.nih.gov), we found that the splicing mutation of c.360+1G>C would be predicted to produce a truncated protein with the initial 120 amino acids the same as wild type GPR143 and an additional 27 aberrant amino acids followed by a premature termination codon (PTC). While the splicing mutation c.659-1G>A would be predicted to produce a truncated protein with the initial 219 amino acids identical to the wild type GPR143 protein and an additional 3 aberrant amino acids followed by a premature termination codon (PTC). Similarly, the duplication mutation of c.43_50dupGACGCAGC (p.L20PfsX25) in exon 1 would be predicted to result in a frame shift and produce a truncated protein with the initial 19 amino acids the same as the wild type GPR143 protein and an additional 5 aberrant amino acids followed by a premature termination codon (PTC). Thus, the NMD surveillance mechanism could be activated by a PTC-containing mRNA. The abnormal mRNA with a PTC would be degraded under the NMD surveillance mechanism or produce a defective truncated protein through escaping the NMD surveillance^16^,^17^.

The missense mutation of c.703G>A (p.E235K) has been reported previously in North American patients with X-linked ocular albinism^7^.However, the clinical phenotypes caused by E235K mutation could be more complicated in North American Patients than in the Chinese patients, because Schnur and colleagues reported that the patient with E235K mutation had periodic alternating nystagmus, photophobia, high myopia, macular hypoplasia, albinotic fundus, iris transillumination, cleft lipand palate^7^, while the Chinese patients carrying with E235K mutation only showed pendular nystagmus, albinotic fundus and macular hypoplasia.

In addition, the patients carrying *GPR143* mutations had macular hypoplasia obtained by OCT, which further supported clinically that congenital nystagmus in these patients sensory defect type and caused by the maldevelopment of the macular or visual pathways.

Mutations in the *GPR143* gene have been reported in Chinese families with nystagmus^5^,^18^,^19^,^20^,^21^,^22^,^23^. However, the previous reports that some patients with isolated X-linked congenital nystagmus^19^,^22^,^23^ were caused by the *GPR143* mutations should be considered carefully, because there were not sufficient clinical data in these reports to support the hypothesis of isolated nystagmus from *GPR143* variants, suggested by Preising *et al.*^24^. Molecular diagnosis and OCT examination would be helpful in distinguishing between OA1 and isolated X-linked congenital nystagmus.

In summary, we investigated five Chinese families with X-linked nystagmus and identified five mutations in *GPR143* including four novel mutations, c.333G>A (p.W111X), c.360+1G>C, c.659-1G>A and c.43_50dupGACGCAGC (p.L20PfsX25), and a previously described mutation of c.703G>A (p.E235K). These mutations expand the mutation spectrum of the *GPR143* gene and contribute to the study of the molecular pathogenesis of *GPR143.*

## Additional Information

**How to cite this article**: Han, R. *et al. GPR143* Gene Mutations in Five Chinese Families with X-linked Congenital Nystagmus. *Sci. Rep.* **5**, 12031; doi: 10.1038/srep12031 (2015).

## Supplementary Material

Supplementary figure 1

## Figures and Tables

**Table 1:** Clinical features of individuals in five Chinese families.

Family	ID#	Patient /carrier	Age	Mutation	Status	CN,XT,HM	VisualacuityOD;OS	Iris hypopigmentation	Fundus	Macular
NYS-17	III:3	carrier	65	c.333G>A	Heterozygous	none	0.8/0.8	none	Normal	Normal
NYS-17	IV:1	patient	39	c.333G>A	Homozygous	CN XT	0.2/0.2	none	hypopigmentation	hypoplasia
NYS-17	IV:3	patient	36	c.333G>A	Homozygous	CN,XT HM	0.1/0.1	none	hypopigmentation	hypoplasia
NYS-17	IV:9	patient	29	c.333G>A	Hemizygous	CN	0.4/0.3	none	hypopigmentation	hypoplasia
NYS-17	V:2	patient	12	c.333G>A	Hemizygous	CN,XT,HM	0.1/0.1	none	hypopigmentation	hypoplasia
NYS-17	V:3	patient	10	c.333G>A	Hemizygous	CN	0.2/0.2	none	hypopigmentation	hypoplasia
NYS-18	III:4	carrier	35	c.360+1G>C	Heterozygous	none	1.0/1.0	none	Normal	Normal
NYS-18	IV:2	patient	7	c.360+1G>C	Hemizygous	CN	0.1/0.1	none	hypopigmentation	hypoplasia
NYS-19	III:6	carrier	37	c.659-1G>A	Heterozygous	none	0.8/0.8	none	Normal	Normal
NYS-19	IV:6	patient	10	c.659-1G>A	Hemizygous	CN	0.3/0.2	none	hypopigmentation	hypoplasia
NYS-20	I:2	carrier	38	c.43_50dup	Heterozygous	none	1.0/0.8	none	Normal	Normal
NYS-20	II:3	patient	8	c.43_50dup	Hemizygous	CN	0.3/0.3	none	hypopigmentation	hypoplasia
NYS-21	III:6	carrier	56	c.703G>A	Heterozygous	none	0.8/0.6	none	Noraml	Normal
NYS-21	IV:3	patient	29	c.703G>A	Hemizygous	CN	0.3/0.4	none	hypopigmentation	hypoplasia

CN: congenital nystagmus.

XT: extropia.

HM: High myopia.
