# Supplementary material for: GPR143 Gene Mutations in Five Chinese Families with X-linked Congenital Nystagmus
Source: Sci Rep. 2015 Jul 10;5:12031. doi: 10.1038/srep12031 (PMC4498220; doi:10.1038/srep12031)
Supplement: Supplementary figure 1 [file srep12031-s1.pdf]

GPR143 Gene Mutations in Five Chinese Families with X-linked Congenital Nystagmus

Ruifang Han[1,2,*], Xiaojuan Wang[3,*], Dongjie Wang[1,2], Liming Wang[2], Zhongfang Yuan[4], Ming Ying[2], Ningdong Li [1,2]

1. Tianjin Medical University, Tianjin, 300070, People’s Republic of China

2. Molecular Genetic Department, Tianjin Eye Hospital, Tianjin Eye Institute, Tianjin Key Lab of Ophthalmology and Visual Science, Tianjin, 300020, People’s Republic of China

3. Ophthalmologic Department, Xuzhou Eye Institute, Xuzhou, Jiangsu Province, 221000, People’s Republic of China

4. Ophthalmologic Department, Jinan Central Hospital Affiliated to Shandong University, Jinan, Shandong Province, 250013, People’s Republic of China

*. Ruifang Han and Xiaojuan Wang contribute equally in this study.

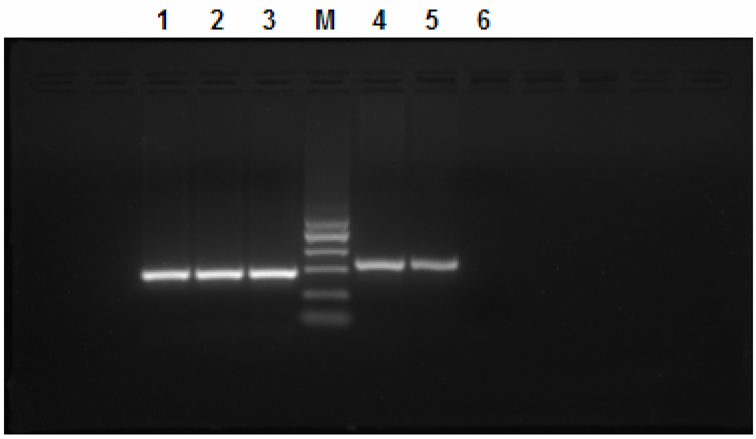

Figure legend

Supplementary figure1. Reverse transcription PCR analysis of *GPR143* mRNA expression in Family NYS-17.

Reverse transcription PCR analysis of *GPR143* mRNA expression in Family NYS-17. The result showed that the *GAPDH* transcripts were detected in a normal control, a female carrier and an affected male (Lane 1, 2, and 3, 192bp size). However, the target product of a 219bp size by reverse transcription PCR amplification from *GPR143* was absent in the affected male (Lane 6), but present in the normal control (Lane 4), and the female carrier (Lane 5). M: the Marker ladder of 25bp, 100bp, 200bp, 300bp, 400bp, 500bp.
